# 2-Methoxyestradiol and its derivatives inhibit store-operated Ca^2+^ entry in T cells: Identification of a new and potent inhibitor

**DOI:** 10.1016/j.bbamcr.2021.118988

**Published:** 2021-05

**Authors:** Anke Löhndorf, Leon Hosang, Wolfgang Dohle, Francesca Odoardi, Sissy-Alina Waschkowski, Anette Rosche, Andreas Bauche, Riekje Winzer, Eva Tolosa, Sabine Windhorst, Stephen Marry, Alexander Flügel, Barry V.L. Potter, Björn-Philipp Diercks, Andreas H. Guse

**Affiliations:** a The Ca^2+^ Signalling Group, Department of Biochemistry and Molecular Cell Biology, University Medical Center Hamburg-Eppendorf, Martinistraße 52, D-20246 Hamburg, Germany; b Institute for Neuroimmunology and Multiple Sclerosis Research, University Medical Centre Göttingen, Von-Siebold-Straße 3a, D-37075 Göttingen, Germany; c Drug Discovery & Medicinal Chemistry, Department of Pharmacology, University of Oxford, Mansfield Road, Oxford OX1 3QT, United Kingdom; d Department of Immunology, University Medical Center Hamburg-Eppendorf, Martinistraße 52, D-20246 Hamburg, Germany; e Department of Biochemistry and Signal Transduction, University Medical Center Hamburg-Eppendorf, Martinistraße 52, D-20246 Hamburg, Germany

**Keywords:** [Ca^2+^]_i_, free cytosolic calcium concentration, 2ME2, 2-methoxyestradiol, ADP, adenosine diphosphate, AM, acetoxymethyl ester, APC, antigen-presenting cell, ATP, adenosine triphosphate, BSA, bovine serum albumin, cADPR, cyclic ADP-ribose, CD, cluster of differentiation, cmp, compound, CNS, central nervous system, E2, estradiol, EAE, experimental autoimmune encephalomyelitis, ER, endoplasmic reticulum, F, fluorescence, G-GECO, green fluorescent genetically encoded Ca^2+^ indicator for optical imaging, GFP, green fluorescent protein, HPLC, high-pressure liquid chromatography, IgG, immunoglobulin G, IL, interleukin, Im, ionomycin, IP_3_, d-*myo*-inositol 1,4,5-trisphosphate, K_d_, dissociation constant, K_V_, voltage-gated potassium channel, MBP, myelin basic protein, MS, multiple sclerosis, NAADP, nicotinic acid adenine dinucleotide phosphate, NCS, newborn calf serum, NFAT, nuclear factor of activated T cells, NMR, nuclear magnetic resonance, PCR, polymerase chain reaction, PLL, poly-l-lysine, ROI, region of interest, SERCA, sarco/endoplasmic reticulum Ca^2+^ ATPase, SOCE, store-operated Ca^2+^ entry, TCR, T cell receptor, TG, thapsigargin, Th1/2, T helper cells type 1/2, T_MBP_, myelin basic protein-reactive CD4^+^ rat T cells, TRPA, transient receptor potential ion channel type A, TRPV, transient receptor potential ion channel type V, ORAI inhibitor, Ca^2+^ signaling, Store-operated Ca^2+^ entry, T cell activation

## Abstract

T cell activation starts with formation of second messengers that release Ca^2+^ from the endoplasmic reticulum (ER) and thereby activate store-operated Ca^2+^ entry (SOCE), one of the essential signals for T cell activation. Recently, the steroidal 2-methoxyestradiol was shown to inhibit nuclear translocation of the nuclear factor of activated T cells (NFAT). We therefore investigated 2-methoxyestradiol for inhibition of Ca^2+^ entry in T cells, screened a library of 2-methoxyestradiol analogues, and characterized the derivative 2-ethyl-3-sulfamoyloxy-17β-cyanomethylestra-1,3,5(10)-triene (STX564) as a novel, potent and specific SOCE inhibitor. STX564 inhibits Ca^2+^ entry *via* SOCE without affecting other ion channels and pumps involved in Ca^2+^ signaling in T cells. Downstream effects such as cytokine expression and cell proliferation were also inhibited by both 2-methoxyestradiol and STX564, which has potential as a new chemical biology tool.

## Highlights

• 2-Ethyl-3-sulfamoyloxy-17β-cyanomethylestra-1,3,5(10)-triene is a SOCE inhibitor.
• novel inhibitor antagonizes SOCE and subsequent signaling events in different T cells
• no antagonist effects on IP_3_ receptor, K_V_ channels or SERCA pumps

## 1 Introduction

Upon T cell receptor (TCR)/CD3-complex and CD28 stimulation, second messengers like nicotinic acid adenine dinucleotide phosphate (NAADP) [1], d-*myo*-inositol 1,4,5-trisphosphate (IP_3_) [2], and cyclic ADP-ribose (cADPR) [3] are formed. These cause a substantial Ca^2+^ release from the ER that leads to globally increased free cytosolic Ca^2+^ concentration ([Ca^2+^]_i_) by both Ca^2+^ release and store-operated Ca^2+^ entry (SOCE) *via* ORAI channels (reviewed in [4,5]). The increased [Ca^2+^]_i_ facilitates binding of Ca^2+^ to calmodulin activating various downstream signaling events, among others translocation of transcription factors into the nucleus.

2-Methoxyestradiol (2ME2) is an endogenous metabolite of estradiol (E2) that is hydroxylated to 2-hydroxyestradiol by cytochrome P450 and subsequently methylated by catechol *O*-methyl transferase (reviewed in [6]). 2ME2 is known for its tumor-inhibiting potential [7,8]. Although 2ME2 originates from estradiol, it shows an at least 500-fold reduced affinity to isolated estrogen receptors [9]. Furthermore, its anti-proliferative behavior is thought to be due to its binding to the colchicine site of tubulin, inhibiting polymerization [10]. Thereby, 2ME2 causes a cell cycle arrest in the G2/M phase during mitosis [11]. Recently, 2ME2 was shown to inhibit proliferation not only in cancer cell lines, but also in T cells thereby ameliorating disease severity in animal models of autoimmune diseases like multiple sclerosis (MS) [12]. In the latter study, 2ME2 blocked translocation of the transcription factor ‘nuclear factor of activated T cells’ (NFAT) into the nucleus of T cells suggesting that 2ME2 interferes with T cell Ca^2+^ signaling.

Here, we investigated 2ME2 regarding inhibition of Ca^2+^ entry in T cells, screened a library of 2ME2 analogues, and characterized STX564 as novel, potent and specific SOCE inhibitor, similar in efficacy to the known SOCE inhibitor Synta66 [13].

## 2 Materials and methods

### 2.1 Reagents

HEPES was purchased from Roth. Synta66 was purchased from Aobious (USA). 2-Methoxyestradiol (2ME2) and its derivatives (STX compounds) were synthesized as described in Table 1, were fully characterized by standard spectroscopic techniques, NMR and mass spectrometry, and their purity assessed as >95% by HPLC. Unless otherwise noted, chemicals were purchased either from Sigma or Merck.

**Table 1:** Literature reference describing compound (cmp) synthesis. The number of the compound (in **bold**) corresponds to that in the relevant publication.

2ME2	Leese et al. [8] (**2**)	STX641	Leese et al. [14] (**15**)
STX49	Leese et al. [15] (**20**)	STX505	Leese et al. [14] (**7**)
STX68	Leese et al. [8] (**4**)	STX564	Leese et al. [14] (**8**)
STX738	Leese et al. [15] (**32**)	STX1175	Jourdan et al. [16] (**46**)
STX139	Bubert et al. [17] (**2**)	STX1177	Jourdan et al. [16] (**47**)
STX243	Leese et al. [15] (**23**)	STX1306	Jourdan et al. [18] (**60**)
STX640	Leese et al. [14] (**14**)	STX1307	Jourdan et al. [18] (**61**)

### 2.2 Culturing of Jurkat T-lymphocytes and human lung carcinoma cell line (H1299)

Jurkat T-lymphocytes were cultured in RPMI 1640 medium containing 25 mM HEPES and GlutaMAX-1 (Gibco, Life Technologies), supplemented with 7.5% (*v*/v) newborn calf serum (NCS) (Biochrom, Merck Millipore), 100 U/ml penicillin and streptomycin (standard JMP medium). Cell density was kept between 0.3 and 1.2 ∗ 10^6^ cells/ml. H1299 cells were cultured in DMEM (Dulbecco's modified Eagle's medium; Gibco, Life Technologies) supplemented with 10% (*v*/v) (FCS) (Biochrom, Merck Millipore), 4 mM l-glutamine, 100 μg/ml streptomycin, and 100 units/ml penicillin.

### 2.3 Isolation of primary human CD4^+^ T cells for Ca^2+^ measurements

Buffy coats were obtained from the blood bank of the University Medical Center Hamburg-Eppendorf (UKE) and peripheral blood mononuclear cells (PBMCs) were isolated by Biocoll density gradient centrifugation (Merck). Human CD4^+^ T cells were isolated from PBMCs by negative selection using the EasySep Human CD4^+^ T Cell Enrichment Kit (Stemcell Technologies) according to the manufacturer's protocol. The purity of the isolated cells was assessed by flow cytometry.

### 2.4 Fura2 loading

Jurkat T-lymphocytes, primary human CD4^+^ T cells and H1299 cells were pelleted at 450 ×*g* for 5 min, rat T_MBP_ at 300 ×*g* for 8 min at room temperature and were resuspended in 1 ml of either standard JMP medium (Jurkat, H1299 and human CD4^+^ T cells) or standard DMEM based medium containing 10% fetal bovine serum (FBS) (*v*/v) (T_MBP_ cells). Resting T_MBP_ cells (day 5–7 after re-stimulation) after the second to fifth re-stimulation were thawed from liquid nitrogen 12 to 40 h prior to Fura2-loading. After pre-incubation for 5 min at 37 °C, cells were incubated with 4 μM Fura2-AM for 15 min at 37 °C. After addition of the dye, cells were kept in the dark. Afterwards, cells and the dye were diluted by addition of 4 ml standard medium and incubated for another 15 min. Cells were washed twice in Ca^2+^ buffer (140 mM NaCl, 5 mM KCl, 1 mM MgSO_4_, 1 mM CaCl_2_, 1 mM NaH_2_PO_4_, 20 mM HEPES, 5.5 mM glucose; pH 7.4 (NaOH), sterile filtered) and were diluted to a concentration of 2*10^6^ cells/ml. Subsequently, cells were kept at room temperature for 20 min for de-esterification before the start of the first measurement.

### 2.5 Ca^2+^ measurements *via* fluorescence spectrophotometry and data analysis

10^6^ Jurkat T-lymphocytes, H1299 cells, T_MBP_ cells or 4*10^6^ primary human CD4^+^ T cells were added into a 10 × 10 mm quartz glass cuvette (Hellma Analytics, Germany) that was placed into a fluorescence spectrophotometer (F-2710, Hitachi, Japan). In experiments using a Ca^2+^ free/Ca^2+^ re-addition protocol, cells were pelleted as described for Fura2 loading and Ca^2+^ buffer was exchanged by 1 ml nominal Ca^2+^ free buffer (140 mM NaCl, 5 mM KCl, 1 mM MgSO_4_, 1 mM NaH_2_PO_4_, 20 mM HEPES, 5.5 mM glucose; pH 7.4 (NaOH), sterile filtered) immediately before starting the measurement. The measurement was controlled by FL Solutions (Version 4.1, Hitachi High-Technologies Corporation). Fura2 was alternatively excited every 2 s at 340 ± 5 nm and 380 ± 5 nm, each 400 ms apart. Fluorescence intensity was measured at an emission wavelength of 495 ± 5 nm for both excitation wavelengths.

Finally, every measurement was calibrated using 0.1% (*v*/v) Triton X-100 to obtain a maximal ratio and 4 mM EGTA and 30 mM Tris to obtain a minimal ratio. The following equation from Grynkiewicz et al. [19] was used to calculate the free cytosolic Ca^2+^ concentration ([Ca^2+^]_i_) from the ratio data:

(1) $${\left[{\mathrm{Ca}}^{2+}\right]}_{i}=K_{d}∙\left(\frac{R-R_{\min}}{R_{\max}-R}\right)∙\left(\frac{F_{380\;\max}}{F_{380\;\min}}\right)$$

*K*_d_ is the dissociation constant for Ca^2+^ and Fura2 which is 224 nM [19]. *R* is the ratio between F_340_ and F_380_. *R*_min_ and *R*_max_ are the minimal and maximal ratio, respectively, obtained by calibration. *F*_*380 min*_ and *F*_*380 max*_ are the minimal and maximal fluorescence intensity at 380 nm EX during the calibration. Finally, artefacts due to compound additions that usually could easily be identified and lasted about 8 s were deleted from the tracings using FL Solutions (Version 4.1, Hitachi High-Technologies Corporation). The fluorescence intensities of deleted data points were interpolated from those just before and after the deleted time points.

Estradiol, 2ME2 and 2ME2 derivatives were dissolved in DMSO at a concentration of 50 mM and stored at −20 °C for up to 6 months. For concentration-response curves, stock solution was freshly diluted with DMSO to obtain the same DMSO concentration of 0.2% (*v*/v) for all conditions.

For calculating IC_50_ values, the mean Ca^2+^ concentrations between 425 s to 475 s after Ca^2+^ re-addition (plateau phase) were calculated and subtracted from the basal Ca^2+^ concentration that represents the mean Ca^2+^ concentration before the first compound addition. These plateau Ca^2+^ concentrations were normalized to 100% (DMSO condition of every data set) and 0% (50 μM STX49 that showed the smallest plateau Ca^2+^ concentrations after Ca^2+^ re-addition). The compound concentrations were then log transformed. Finally, the data were fitted to a corresponding nonlinear fit of variable slope using Prism 6 (GraphPad Software, USA).

### 2.6 Mn^2+^ quenching of Fura2

Mn^2+^ quenching of Fura2 was used to analyze influx of divalent cations into Jurkat T cells. Like for Ca^2+^ measurements, 10^6^ loaded Jurkat T-lymphocytes were pelleted and Ca^2+^ buffer was exchanged for nominal Ca^2+^ free buffer (see above). Instead of Ca^2+^, Mn^2+^ was added 1220 s after the start of the measurement. To investigate Mn^2+^ quenching independently of any effects of Ca^2+^, Fura2 was excited at its isosbestic point, which was experimentally determined to be 357 nm. The emission wavelength was set to 495 nm. In contrast to the Ca^2+^ measurement experiments, these measurements were not calibrated.

For quantitative analysis, the velocity of quenching was calculated as the initial slope. After Mn^2+^ addition, there was a very fast drop in Fura2 fluorescence in all experiments. This drop lasted for about 10 s and was probably due to quenching of extracellular Fura2. This phase was not considered in the calculation of the initial slope. The data after this rapid drop were fitted to a one phase exponential decay with the following equation:

(2) y=(y0–P)*e(-K*x)+P

*y*_*0*_ is the fluorescence at the start of the decay, *P* is the theoretical fluorescence at the end of the decay at infinite times, *K* is the rate constant, and *x* is the time.

The first derivative of this equation was used to calculate the slope at 1230 s:

(3) y=(y0–P)*(-K)*e(-K*x)

In all experiments, fluorescence gradually decreased before Mn^2+^ addition due to bleaching. The fluorescence before Mn^2+^ addition was fitted to a straight line using the following equation:

(4) y’=m·x+c

*m* is the slope, *x* is the time and *c* is the y-intercept. To not overestimate the initial slope, the slope of this linear fit was subtracted from the initial slope of the exponential decay. So the initial slope (s_i_) after Mn^2+^ addition was calculated using the following equation:

(5) si=(y0–P)*(-K)*e(-K*1230s)–m

### 2.7 G-GECO1.2-ORAI1 transfection of Jurkat JMP T-lymphocytes and live cell imaging

12.5 ∗ 10^6^ Jurkat T–lymphocytes were pelleted at 400 to 450 ×*g* for 5 min at room temperature and pellets were dissolved in 500 μl RPMI 1640 medium (Gibco) without phenol red containing l-glutamine, supplemented with 7.5% (*v*/v) NCS. 30 μg plasmid-DNA (G-GECO1.2-ORAI1) was transferred into a 4 mm electroporation cuvette (Biozym, Austria) and the Jurkat T-lymphocytes were added. G-GECO1.2-ORAI1 was a gift from Michael Cahalan, USA (Addgene plasmid # 73562) [13]. After an incubation of 5 min at 37 °C, cells were electroporated with 960 μF and 240 V (τ: 14.7 to 16 s, mean: 15.3 s). After an additional incubation for 10 min in the incubator, the cells were transferred into a cell culture flask containing 10 ml standard JMP medium.

17 to 22 h after electroporation, 5 ∗ 10^4^ cells were pelleted at 400–450 ×*g* for 5 min at room temperature and cell pellets were dissolved in 50 μl nominal Ca^2+^ free buffer. Cells were placed on coverslips freshly coated firstly with 5 μl of 5 mg/ml BSA and subsequently with 5 μl of 0.2 μg/ml PLL. The cell chamber was constructed using a rubber O-ring attached to the coverslip with medium viscous silicone (Baysilone, Bayer). Cells were allowed to settle down for 5 min at room temperature. Subsequently, 50 μl fresh nominal Ca^2+^ free buffer was added and the coverslip with cells was placed onto an inversed Leica microscope (type DM IRBE). Those cells were selected which showed an intermediate and equally distributed fluorescence throughout the outer rim (plasma membrane). The fluorophore was excited at 488 nm every 2 s for 30 min and fluorescence was measured at 520 nm using Volocity (Version 6.3, Perkin Elmer).

Using ImageJ (Version 1.51 k, National Institutes of Health, USA) the pictures were analyzed for mean fluorescence in the outer ring of Jurkat T-lymphocytes employing ring-shaped regions of interest (ROIs). Only those cells that showed an increase in fluorescence after ionomycin addition were analyzed. For background correction, three to four ROIs were placed in areas with no cells. The mean background fluorescence values were subtracted from the signal in every image. For quantitative analysis, fluorescence intensities were normalized to the first fluorescence intensity (F/F_0_).

### 2.8 Cell permeabilization and data analysis

3 ∗ 10^7^ Jurkat T-lymphocytes per measurement were centrifuged at 450 ×*g* for 5 min at room temperature. The pellet was rinsed twice, each time using 10 ml permeabilization buffer 1 (120 mM KCl, 10 mM NaCl, 1.2 mM MgCl_2_, 0.533 mM CaCl_2_, 1 mM EGTA and 10 mM HEPES, pH 7.2 (KOH), sterile filtered). Subsequently, the pellet was resuspended in 2 ml permeabilization buffer 1 containing 80 μg/ml saponin (Fluka) and the cells were incubated for 10 min at 37 °C. Subsequently, the cells were centrifuged at 450 ×*g* for 5 min at room temperature and the cell pellet was washed once in 10 ml permeabilization buffer 2 (120 mM KCl, 10 mM NaCl, 1.2 mM MgCl_2_ and 10 mM HEPES, pH 7.2 (KOH), sterile filtered). Finally, the cells were taken up in 1 ml permeabilization buffer 2, placed into a cuvette and measured at the fluorescence spectrophotometer. Before the start of the measurement, 0.5 μl of 1 mM Fura2 free acid (dissolved in pure water, final concentration: 0.5 μM), 5 μl of 5 mg/ml creatinkinase (dissolved in permeabilization buffer 2, final concentration: 25 μg/ml, 20 U/ml, Roche) and 10 μl of 2 M creatin phosphate (dissolved in permeabilization buffer 2, final concentration: 20 mM, Calbiochem) were added. Fura2 was used to measure the Ca^2+^ concentration and creatinkinase and creatin phosphate were used as ATP-regenerating system. The measurement was controlled by FL Solutions (Version 4.1, Hitachi High-Technologies Corporation). Fura2 was alternatively excited every 2 s at 340 ± 5 nm and 380 ± 5 nm, each 400 ms apart. Fluorescence intensity was measured at an emission wavelength of 495 ± 5 nm for both excitation wavelengths. Finally, every measurement was calibrated by adding 2 mM CaCl_2_ and 0.1% (*v*/v) Triton X-100 to obtain a maximal ratio and, to obtain a minimal ratio, by 4 mM EGTA and 30 mM Tris. Ca^2+^ concentrations were calculated according to Grynkiewicz et al. [19] as described earlier in Section 2.4.

As a quantitative read-out for inhibition of IP_3_ receptors, the peak [Ca^2+^] after IP_3_ addition was subtracted from the mean [Ca^2+^] between 25 and 75 s before IP_3_ addition (dPeak). For analyzing SERCA inhibition, the data after compound addition, namely thapsigargin, STX564, Synta66 or DMSO as a vehicle control, and the decay after ATP addition was fitted to a ‘plateau followed by one phase exponential decay’ using GraphPad Prism. The rate constant K of the fitted data was used as a quantitative read-out for SERCA pump activity and its inhibition.

### 2.9 Electrophysiology

For whole-cell patch-clamp measurements [20], 35 mm cell culture dishes (Greiner Cellstar, Germany) were coated with 0.01% (*w*/*v*) PLL solution (150,000–300,000 g/mol) diluted 1:50 with high-purity water (final concentration: 2 μg/ml) for 30 min. Coated culture dishes were rinsed twice with high-purity water, dried at room temperature under sterile conditions and then stored at 4 °C for up to 1 week. Just before starting the experiments, coated culture dishes were pre-warmed for 5 min at room temperature. A recording chamber (RC-37F or RC-37 W, Warner Instruments, USA) that was preliminary coated with a thin layer of baysilone-paste (GE Bayer silicones, Germany) of medium viscosity was placed into the coated cell culture dish. About 10^5^ Jurkat T-lymphocytes were placed into this recording chamber and were allowed to settle down for 5–10 min. Afterwards the cells were rinsed twice with 200 μl extracellular buffer (composition: see below next paragraph) and finally taken up in 200 μl extracellular buffer containing 200 nM thapsigargin as well as either K^+^ channel inhibitors, STX compounds or 0.2% (*v*/v) DMSO as a vehicle control. The cell culture dish was placed in a custom-made holder placed on an inverted microscope (Zeiss AxioVert 100). Microelectrodes were manually pulled from 1.5 mm diameter borosilicate glass capillaries (Science Products, Germany) and firepolished *via* a Flaming Brown micropipette puller (model P-97; Sutter instrument Co., USA), resulting in pipette resistances between 2.7 and 5.1 MΩ (mean: 3.9 MΩ). The microelectrodes were filled with intracellular solution (composition: see below next paragraph). The holding potential was set to −60 mV. Extracellular buffer for K_V_ measurement contained 128 mM CH_3_SO_3_Na, 4 mM NaCl, 2 mM CaCl_2_, 1 mM MgCl_2_, 10 mM HEPES, 10 mM glucose; pH 7.4, NaOH (modified from [21]).

Intracellular buffer for measuring K_V_ channels contained 125 mM aspartic acid, 5 mM MgCl_2_, 2 mM MgSO_4_, 15 mM HEPES, 12 mM BAPTA (pH 7.2, KOH, sterile filtered; modified from [21]). For measuring K_V_ channels, a step protocol was started ranging from +80 to −80 mV with an intersweep interval of 30 s at the holding potential to circumvent inactivation. The voltage was applied for 100 ms and was decreased by 10 mV after every sweep. Data were low-pass filtered at 1 kHz and fast and slow capacity currents (c-fast and c-slow) were compensated by Patchmaster (HEKA, Germany). C-slow and series resistance was compensated before every sweep. The voltage was applied to the cells and the currents were recorded *via* an EPC9/10 amplifier at a sampling rate of 20 kHz.

### 2.10 Generation and culturing of T_MBP_ cells

Myelin basic protein (MBP)-reactive CD4^+^ T cells retrovirally transduced to express enhanced GFP (T_MBP_ cells) were established as previously reported [22]. In brief, 6–8 week old female Lewis rats were immunized subcutaneously with 150 μl of emulsion consisting of equal parts of MBP (stock concentration: 1 mg/ml) and complete Freund's adjuvant containing heat-inactivated *Mycobacterium tuberculosis* extract (Difco; stock concentration: 2 mg/ml). Draining lymph nodes were harvested and passed through a cell strainer (40 μm) 9–10 days after immunization. The cell suspension was co-cultured with GP + E86 packaging cell lines producing replication-deficient retroviruses in the presence of 10 μg/ml MBP. Murine IL-2 was added to the culture for T cell expansion from day 2–4 after stimulation. The cell lines underwent 3–4 cycles of stimulation before being used for *in vitro* and *in vivo* experiments. T-cell lines were CD4^+^, CD8^−^, αβTCR^+^ and displayed an effector memory phenotype (L-selectin^−^, CD45RC^low^, CD44^high^). Upon stimulation, they produced interferon-γ and IL-17.

### 2.11 *In vitro* activation assay of T_MBP_ cells

*In vitro* activation assays were performed in 96-well plates. Briefly, 10^5^ T_MBP_ cells and 10^6^ 30 Gy-irradiated thymocytes per well were co-cultured in presence of in 10 μg/ml MBP and varying concentrations (0.01, 0.1, 1, 10, 100 μM) of 2ME2, STX564 or Synta66 dissolved in DMSO. The working concentration of DMSO/well was 0.02% (*v*/v). T_MBP_ cell activation on mRNA level was determined using qPCR and on surface protein level using surface marker staining and subsequent flow cytometry analysis.

### 2.12 *In vitro* activation assay of human PBMCs

*In vitro* activation assays were performed in 96-well plates. Briefly, 10^5^ PBMCs isolated from human peripheral blood by Ficoll gradient were added onto plates coated with anti-human CD3 monoclonal antibody (5 μg ml^−1^ in PBS, clone OKT3, Biolegend). 2ME2 or STX564 dissolved in DMSO were added in varying concentrations (0.01, 0.1, 1, 10 μM). The working concentration of DMSO/well was 0.02% (*v*/v). PBMC activation on mRNA level was determined using qPCR on samples collected 24 h after CD3 stimulation.

### 2.13 Cell isolation and flow cytometry

To assess *in vitro* T cell activation, surface staining for CD25 (OX39, Bio-Rad) was performed. APC-labelled goat anti-mouse antibody (Jackson ImmunoResearch) was used as secondary antibody. Mouse IgG1κ (MOPC 31C, Sigma-Aldrich) was used as control.

Cytofluorometric analysis was carried out with a CytoFLEX S (Beckman Coulter) operated by CytExpert software (Beckman Coulter). All procedures were performed at 4 °C. Data were analyzed by using FlowJo software (FlowJo, LLC).

### 2.14 Quantitative PCR

Total RNA was isolated using the standard TRIzol method. cDNA was synthesized using the RevertAid First Strand cDNA Synthesis Kit (Thermo Fisher Scientific). Quantitative PCR (qPCR) was performed using the StepOnePlus™ Real-Time PCR System (Applied Biosystems), as previously described [[23], [24], [25]]. Rat T_MBP_ cell data were obtained from three to four independent duplicate measurements. β-actin served as housekeeping gene. Human PBMC data were obtained from two independent healthy donors and duplicate measurements (representative data of one donor are shown). HPRT served as housekeeping gene. For all probes (TaqMan), a combination of 5′-FAM and 3′-TAMRA quencher was used as described [26,27].

### 2.15 Statistics

Data analysis was performed with Excel (Microsoft, USA) and Prism (GraphPad Software, USA). Data sets were tested for normal distribution by the Kolmogorov-Smirnov test with Dallal-Wilkinson-Lillie for *P* value. Normally distributed data were analyzed *via* unpaired two-tailed *t-*test or ANOVA and Bonferroni correction for multiple testing. If the data were not normally distributed, a non-parametric Kruskal-Wallis test with Dunn's correction for multiple testing was performed. The latter was also performed if the sample size was too small to be checked for normality by the Kolmogorov-Smirnov test. For statistical testing, a significance level of α = 0.05 was adopted. An *a priori* power analysis to determine sample size was not performed.

## 3 Results

### 3.1 2ME2 derivative STX564: a high affinity and highly selective antagonist of SOCE

**Fig. 1:**
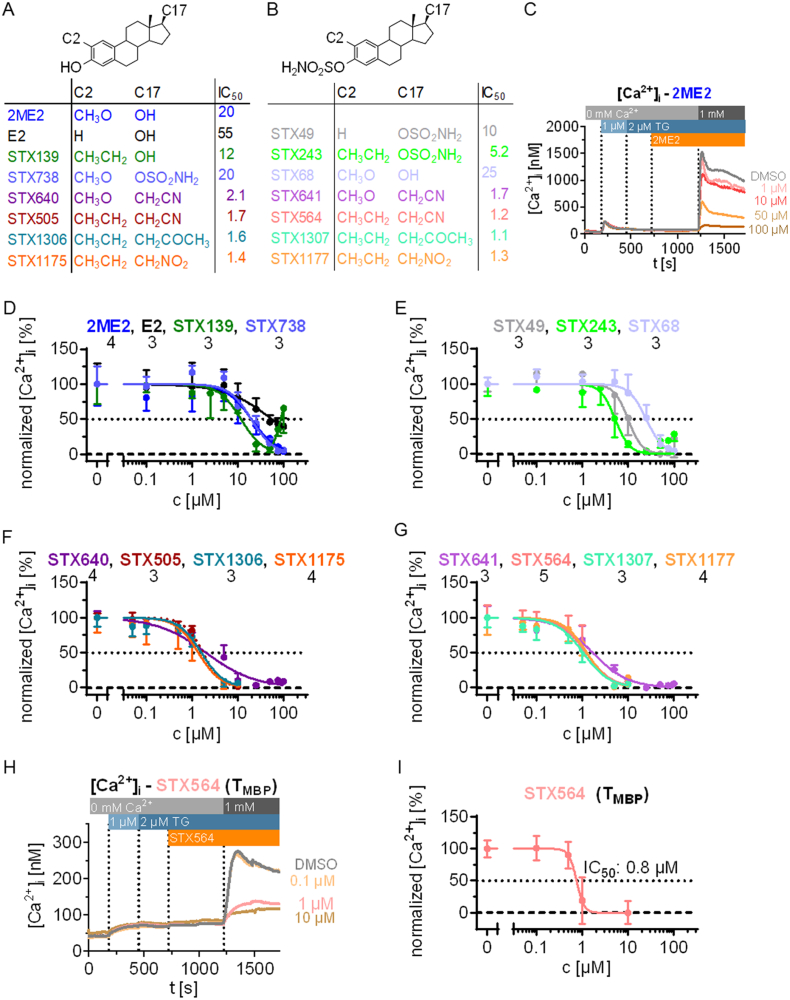
2-Methoxyestradiol (2ME2), estradiol (E2), and synthetic 2ME2-derivatives inhibit SOCE in human Jurkat T cells and primary rat T_MBP_ cells. A, B: Structures and IC_50_ values of SOCE inhibition of 2ME2, E2 and synthetic 2ME2-derivatives. C: Example of the Ca^2+^ free/Ca^2+^ re-addition protocol showing Ca^2+^ tracings of Jurkat T cells and inhibition of SOCE by different 2ME2 concentrations (mean data, *n* = 4 independent measurements). D-G: Concentration-response curves of the different compounds (mean ± SD, n indicated below compound names). These concentration-response curves were used to calculate the IC_50_ values shown in A and B. H: Ca^2+^ free/Ca^2+^ re-addition protocol showing Ca^2+^ tracings of primary rat T cells and inhibition of SOCE by different STX564 concentrations (mean data, *n* = 3 independent measurements). I: Concentration-response curve of STX564 treated primary rat T cells (mean ± SD, n = 3 measurements).

2ME2 inhibits Ca^2+^ dependent translocation of NFAT in T cells effectively and specifically [12] indicating that it interferes with the upstream Ca^2+^ signaling cascade. We screened a library of 2ME2 and synthetic 2ME2 derivatives (Fig. 1A,B) regarding inhibition of SOCE in human Jurkat T cells using a Ca^2+^ free/Ca^2+^ re-addition protocol where test compounds were added to cells with their Ca^2+^stores fully depleted in nominally Ca^2+^ free medium and SOCE was quantified upon re-addition of 1 mM extracellular Ca^2+^ (Fig. 1C). Since the IC_50_ for 2ME2 was quite high (20 μM, Fig. 1A,C,D), several synthetic derivatives structurally modified at either C2 or C17 or at both sites were analyzed for comparison (Fig. 1D-G). The synthetic compound 2-ethyl-estradiol (STX139) differs from 2ME2 by the substitution of the 2-methoxy group by a 2-ethyl group (Fig. 1D) and exhibits an IC_50_ of about 12 μM. An almost complete inhibition of Ca^2+^ entry was obtained at 50 μM. With these specifications, STX139 is almost twice as potent as 2ME2, suggesting that a more hydrophobic 2-ethyl group increases compound potency in a similar way to a 2-methoxyl group. A sulfamoyloxy group [30,31] at either C3 (STX68) or C17 (STX738) exhibits similar IC_50_ values (STX68: 25 μM; STX738: 20 μM; Fig. 1D,E), indicating that the group itself is not crucial for decreasing Ca^2+^ entry *in vitro*. The same is true for the 3,17β-bis-sulfamoyloxylated derivative of STX139, namely STX243. The IC_50_ is 5.2 μM (Fig. 1E) and is thus approx. 2-fold smaller as compared to STX139. An almost complete Ca^2+^ entry inhibition is obtained at 25 μM. Unexpectedly, 3,17β-bis-sulfamoyloxy-estradiol (STX49) [28] shows greatly increased Ca^2+^ entry inhibition compared to estradiol with an IC_50_ of 10 μM (Fig. 1E). However, this compound is somewhat less potent than STX243.

These results slightly favour the 2-ethyl compounds over the 2‑hydrogen or 2-methoxyl compounds as new and potent targets for drug development. Except for STX49, which differs in its potency from its 3,17β-bis-hydroxyl compound estradiol, 3,17β-bis-sulfamoyloxyl compounds do not differ much in their IC_50_ values from their 3,17β-hydroxylated compounds. Despite this, these compounds are more suitable for animal experiments as they show a higher bioavailability [29].

The 2-methoxy-17β-cyanomethyl compounds STX640 (3-hydroxyl) and STX641 (3-sulfamoyloxy) exhibit an IC_50_ of about 2.1 μM and 1.7 μM, respectively (Fig. 1F,G). These compounds are 10-fold more potent than the 17β-hydroxyl-compound (2ME2) suggesting that the substitution at C17β to cyanomethyl increases the potency of Ca^2+^ entry inhibition.

As compounds possessing a 2-ethyl moiety were more potent in inhibiting Ca^2+^ increase, the 2-ethyl-17β-cyanomethyl compounds (STX505, STX564) were also evaluated in the Ca^2+^ free/Ca^2+^ re-addition protocol. Interestingly, they did not differ from the 2-methoxy compounds STX640 and STX641, respectively, concerning their IC_50_ values (STX505: 1.7 μM; STX564: 1.2 μM) for inhibiting Ca^2+^ entry (Fig. 1F,G). Nevertheless, they showed a highly increased potency in Ca^2+^ entry inhibition compared to 17β-hydroxyl or 17β-sulfamoyloxyl compounds. Compounds with further substitutions at the C17β position to nitromethyl (STX1175, STX1177) or oxopropyl groups (STX1306, STX1307) exhibit similar IC_50_ values as the 17β-cyanomethyl compounds (Fig. 1F,G).

These results demonstrate that substitution at the C17β position by cyanomethyl, nitromethyl or oxopropyl groups does increase the potency of 2ME2 derivatives, but it is not important which specific substitution is made. These side groups provide a sterically unhindered hydrogen bond acceptor which may provide a tentative rationale. The sulfamoyloxy substitution provides both hydrogen bond donor and acceptor groups.

Among the three best antagonists, STX564, STX1307, and STX1177 with IC_50_ values close to 1 μM, STX564 was chosen for further characterization. In order to confirm the effects of STX564 in non-transformed T cells, we employed rat T cells reactive for myelin basic protein (T_MBP_ cells), known to induce experimental autoimmune encephalomyelitis (EAE), a model for multiple sclerosis. Importantly, SOCE in these T_MBP_ cells was effectively inhibited upon incubating with STX564 (IC_50_ 0.8 μM; Fig. 1H,I).

**Fig. 2:**
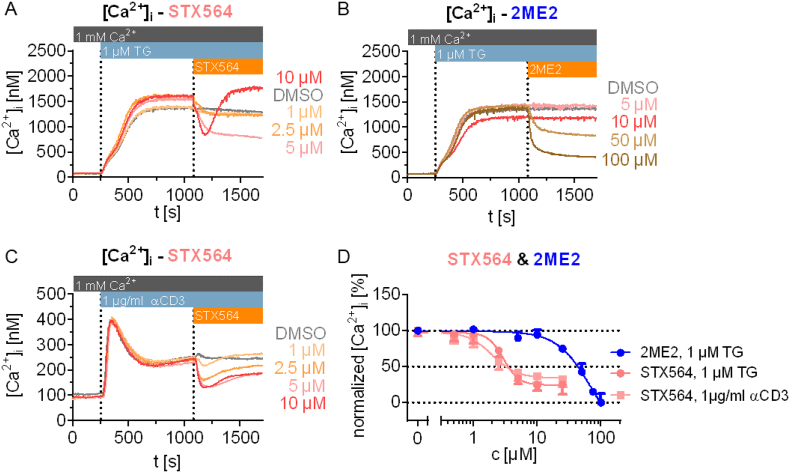
STX564 and 2ME2 immediately inhibit SOCE. Jurkat T cells loaded with Fura2 were stimulated by thapsigargin (TG, A, B) or anti-CD3 monoclonal antibody OKT3 (C) in extracellular buffer containing 1 mM Ca^2+^. Shown are mean tracings of Ca^2+^ signals (*n* = 3 (2ME2, STX564, 1 μM TG) or 4 (STX564, αCD3)). D: Concentration-response curve of 2ME2 and STX564 from the data obtained in A-C (mean ± SD, n as indicated above). The IC_50_ values are for 2ME2 71 μM, for STX564, 1 μM TG 3 μM and for STX564, 1 μg/ml αCD3 2 μM.

Besides the Ca^2+^ free/Ca^2+^ re-addition protocol, we also characterized the effect of STX564 in cells during activated SOCE in the presence of thapsigargin (TG, Fig. 2A,B) or upon stimulation *via* their TCR/CD3 complex (Fig. 2C). Under such conditions, inhibition was effective acutely upon drug addition (Fig. 2). However, only partial inhibition was achieved (Fig. 2D). Two additional aspects of addition of STX564 during active SOCE should be mentioned: Fig. 2A shows that at 10 μM STX564 the initial inhibition of SOCE was rapidly reverted into an even higher steady-state Ca^2+^ entry level. Similarly, in Fig. 2C this effect of a partial or full recovery of SOCE was also observed, a few minutes after STX564 addition.

**Fig. 3:**
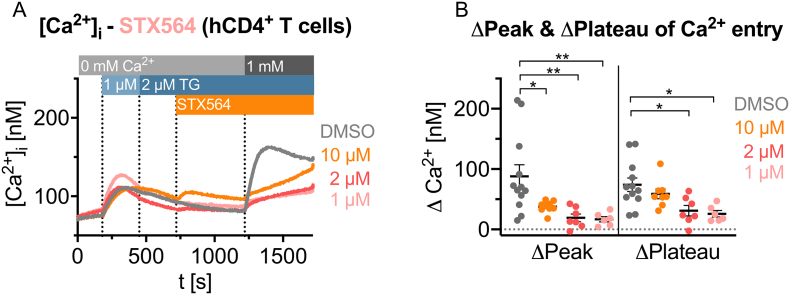
STX564 inhibits SOCE in primary human CD4+ T cells. **A** Mean Ca^2+^ tracings of primary human CD4+ T cells using the Ca^2+^ free/Ca^2+^ re-addition protocol and inhibition of SOCE by different STX564 concentrations (mean data, *n* = 6–12 independent measurements). **B** Analysis of the ΔCa^2+^ peak (1372s) and ΔCa^2+^ plateau (1700 s) after Ca^2+^ re-addition of the data shown in A. Individual data points represents single measurements of 12 (DMSO), 8 (10 μM STX564), 7 (2 μM STX564) or 6 (1 μM STX564) measurements with mean ± SEM. Statistical analysis was performed by comparing the DMSO control with all other conditions with an ordinary ANOVA with Bonferroni correction for multiple testing (*: *p* < 0.05, **: *p* < 0.01).

Since Jurkat T cells are an autonomously growing T-lymphoma cell line, SOCE in primary human T cells was also analyzed regarding potential inhibition by STX564. In these experiments, very low concentrations of STX564 most efficiently inhibited SOCE (Fig. 3). At 1 and 2 μM STX564 both the initial Ca^2+^ peak after re-addition of extracellular Ca^2+^ as well as Ca^2+^ entry at later stages were efficiently inhibited (Fig. 3). In contrast, at 10 μM STX564 the steady-state plateau phase of Ca^2+^ entry was re-reached after a few min, similar to the effects seen in Jurkat T cells in Fig. 2. Also, at 10 μM STX564, Ca^2+^ release was observed directly upon drug addition in the absence of extracellular Ca^2+^ (Fig. 3A). Collectively, the results in Fig. 2, Fig. 3 indicate that STX564 in addition to its antagonist effect on SOCE may, at elevated concentrations, also have an agonist effect on Ca^2+^ release, as shown previously for other SOCE inhibitors [30].

**Fig. 4:**
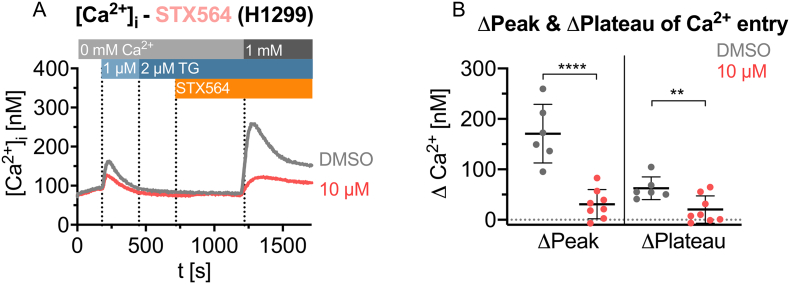
STX564 inhibits SOCE in primary human CD4^+^ T cells. **A** Mean Ca^2+^ tracings of H1299 cells using the Ca^2+^ free/Ca^2+^ re-addition protocol and inhibition of SOCE by 10 μM STX564 (mean data, n = 6–8 independent measurements). **B** Analysis of the ΔCa^2+^ peak (1290s) and ΔCa^2+^ plateau (1680 s) after Ca^2+^ re-addition of the data shown in A. Individual data points represents single measurements of 6 (DMSO) and 8 (10 μM STX564) measurements with mean ± SEM. Statistical analysis was performed by comparing the DMSO control with all other conditions with an ordinary ANOVA with Bonferroni correction for multiple testing (**: p < 0.01, ****: *p* < 0.0001).

Further, we also applied STX564 to a non-T cell known to utilize SOCE during Ca^2+^ signaling, too. Nonsmall cell lung cancer (NSCLC) model cell line, H1299, was subjected to the Ca^2+^ free/Ca^2+^ re-addition protocol as in Fig. 1. As shown in Fig.4, SOCE was also significantly inhibited by STX564 in H1299 cells demonstrating that the drug target is not T cell-specific.

### 3.2 Characterization of the molecular target of STX564

**Fig. 5:**
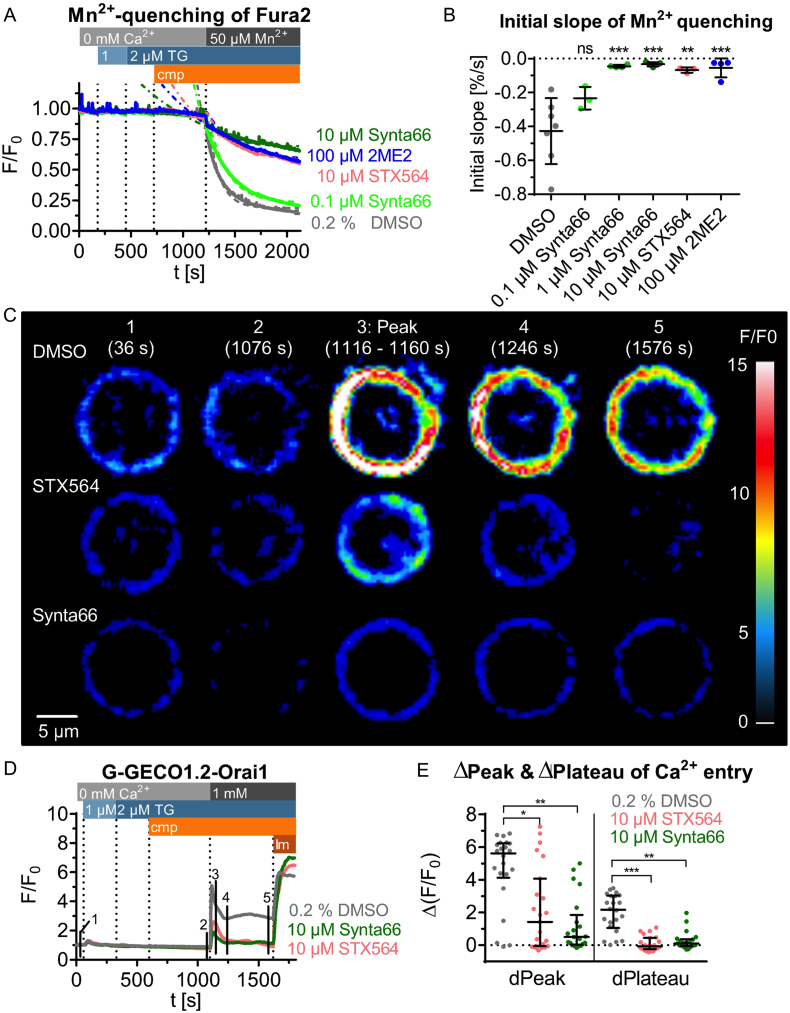
STX564 decreases Ca^2+^ entry by inhibiting ORAI1 channels. A–B: SOCE evoked Mn^2+^ quenching of Fura2 in Jurkat T cells. Store depletion was activated by two subsequent additions of thapsigargin (TG), before compounds (cmp) and Mn^2+^ were added stepwise. The decrease of Fura2 fluorescence after Mn^2+^ addition was fitted to an exponential one phase decay equation (colored dotted lines). The slope of this decay was subtracted by the slope of a straight line fitted to the data before Mn^2+^ addition and is displayed in B. Statistical analysis was performed by comparing the DMSO control with all other conditions with an ordinary ANOVA with Bonferroni correction for multiple testing. ns: not significant, **: *p* < 0.01, ***: *p* < 0.001. Dots represent individual experiments and lines symbolize mean values of 3 (0.1 μM Synta66; STX564), 4 (1 μM Synta66; 2ME2), 5 (10 μM Synta66) or 7 (DMSO) measurements. C–E: G-GECO1.2-ORAI1 transfected Jurkat T cells were investigated by live cell Ca^2+^ imaging. C: Characteristic examples of cells treated with DMSO, STX564 or Synta66 (each at 10 μM). These images were processed by deconvolution, using a 60% reduction of stray light at a gain of 6. D: Mean tracings of the different conditions (n see below). The numbers in the tracings represent different timepoints from which the images in C were taken. E: Quantitative analysis of the data shown in C–D. Individual data points represent single cells and the median ± IQR is indicated by lines. Data were collected from 7 independent transfections which resulted in 21 (Synta66), 22 (DMSO) or 23 (STX564) cells analyzed. Statistical analysis was performed by Kruskal-Wallis test with Dunn's correction. *: *p* < 0.05, **: p < 0.01, ***: p < 0.001.

In addition to the Ca^2+^-free/Ca^2+^ re-addition protocol, entry of divalent ions into Jurkat T cells upon Ca^2+^ store depletion was measured by Mn^2+^ quenching of Fura2 fluorescence (Fig. 5A,B). STX564 antagonized Mn^2+^ entry almost fully at 10 μM; ORAI1 inhibitor Synta66 showed a similar result (Fig. 5A,B) indicating that the SOCE component blocked by STX564 might be ORAI channels. To identify more precisely the target of STX564, transiently transfected G-GECO1.2-ORAI1 Jurkat T-lymphocytes were employed. These cells are stably transfected with a cDNA coding for ORAI1 fused to a genetically encoded low-affinity Ca^2+^ sensor; these cells can be used to exclusively monitor Ca^2+^ fluxes through ORAI1 [13]. STX564 as well as Synta66 (both at 10 μM) almost completely diminished SOCE by ORAI1 (Fig. 5C-E).

Relative fluorescence intensities (F/F_0_ ratio) were significantly decreased compared to vehicle controls (Kruskal-Wallis test, Dunn's corrected, peak: STX564: *p* = 0.012, Synta66: *p* = 0.002; plateau: STX564: *p* < 0.001, Synta66: *p* = 0.003). These results suggest that the SOCE component inhibited by STX564 either is ORAI or its activators STIM1 and STIM2.

**Fig. 6:**
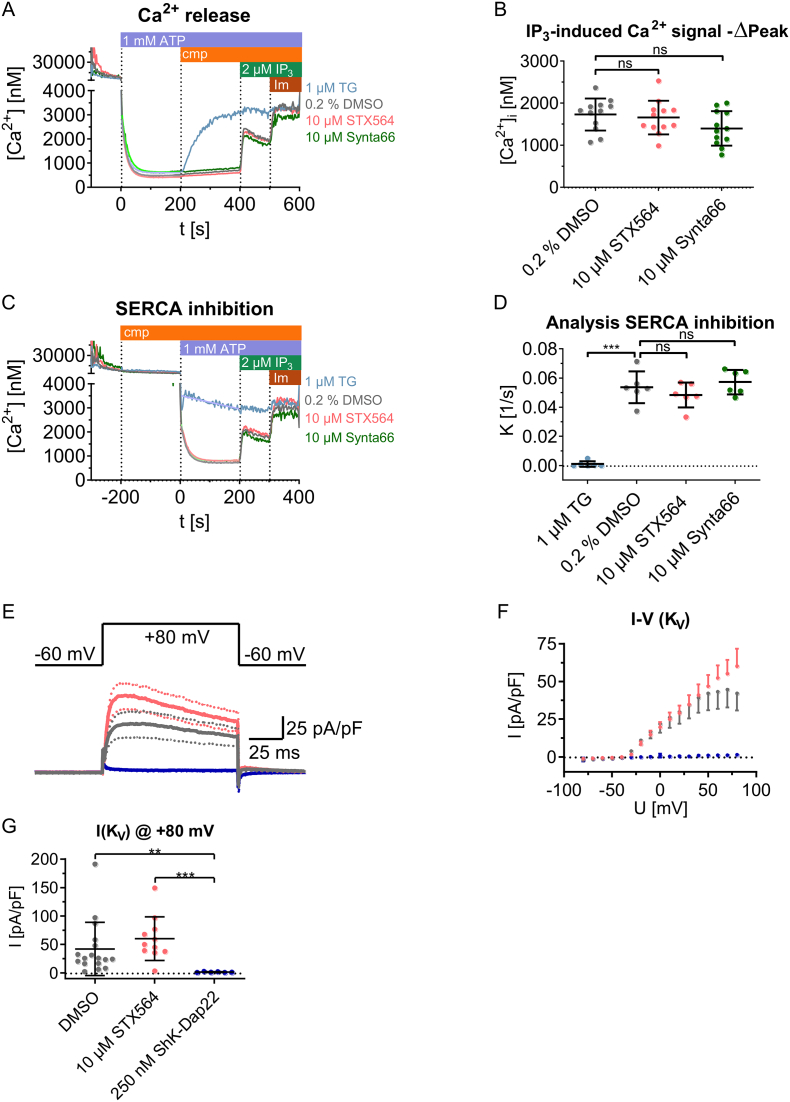
STX564 does neither affect Ca^2+^ release from or Ca^2+^ reuptake into ER nor K_V_ channels. A-D: IP_3_-evoked Ca^2+^ release or SERCA activity was analyzed in permeabilized Jurkat T cells. A–D: Jurkat T cells were permeabilized by saponin, transferred into an intracellular medium, and ER Ca^2+^ stores were re-loaded using ATP and an ATP-regenerating system consisting of creatine phosphate and creatine kinase. Fura2 free acid was added to monitor the free Ca^2+^ concentration in the cuvette. A-B: Upon completion of Ca^2+^ store loading by ATP, SOCE antagonists (cmp) were added, followed by IP_3_. Neither STX564, Synta66 nor vehicle DMSO evoked Ca^2+^ release on their own. These compounds also did not inhibit the Ca^2+^ release by IP_3_. Mean data (A) or mean ± SD (B) values of 12 independent measurements are shown. C–D: To analyze any effects on SERCA activity, SOCE antagonists (cmp) were added before ATP. Neither STX564, Synta66 nor vehicle DMSO affected ATP-induced Ca^2+^ reuptake by SERCA pumps, analyzed as initial negative slope of [Ca^2+^]. Mean data (C) or mean ± SD (D) values of 6 independent measurements are shown. E–G: STX564 does not inhibit K_V_ channels. Mean ± SD currents of K_V_ channels evoked by a step protocol ranging from −80 to +80 mV using whole-cell patch clamp recordings. Mean values were calculated for 17 (DMSO), 11 (STX564) or 6 (SHK-Dap22) cells from 3 (ShK-Dap22) or 4 (DMSO, STX564) independent experiments. STX564 does not significantly differ from DMSO control measurements (Kruskal-Wallis-test, Dunn's correction, *p* = 0.3107), while Kv currents were inhibited by ShK-Dap22 (Kruskal-Wallis-test, Dunn's correction, *p* = 0.0073).

Despite this clear-cut result with G-GECO1.2-ORAI1, we further intended to rule out any additional effects of STX564 on other targets that directly or indirectly may inhibit SOCE. Since decreased SOCE may also be a result of decreased release of Ca^2+^ from intracellular stores, this was analyzed directly in permeabilized Jurkat T cells (Fig. 6A,B). Ca^2+^ stores were initially re-loaded by addition of 1 mM ATP and STX564, or Synta66, or vehicle were added, followed by addition of 2 μM IP_3_, and finally by 1.4 μM ionomycin (Im, Fig. 6A). Neither STX564 nor Synta66 affected IP_3_-evoked Ca^2+^ release significantly, while thapsigargin emptied the Ca^2+^ stores by SERCA inhibition thereby eliminating effects of IP_3_ (Fig. 6B).

Next, we analyzed the effect of STX564 or Synta66 on SERCA activity, using the same permeabilized cell model as before (Fig. 6C,D). SERCA activity was quantified as (negative) initial slope of [Ca^2+^]_i_ upon ATP addition. While thapsigargin almost completely abolished SERCA activity, neither STX564 nor Synta66 showed any effect (Fig. 6D). Further, K_V_ channels are important for T-lymphocyte activation since they partially equilibrate the transfer of positive charges of Ca^2+^ into the cytosol during Ca^2+^ entry by efflux of positively charged K^+^ ions [31,32]. Patch clamp recordings using standard protocol for K_V_ channels revealed no inhibition by 10 μM STX564 while the standard K_V_ channel inhibitor ShK-Dap22 almost completely blocked the current (Fig. 6E-G).

### 3.3 Effects of STX564 on downstream signaling events in CNS-reactive T cells

**Fig. 7:**
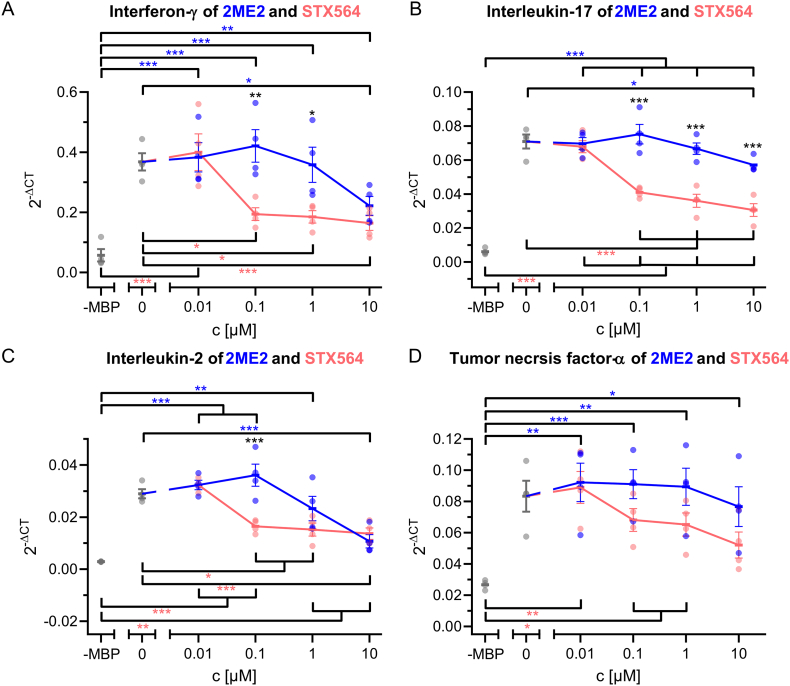
STX564 inhibition of ORAI1 blocks cytokine production and antigen dependent T cell activation. T_MBP_ cells were challenged *in vitro* by the cognate antigen myelin-basic protein (MBP) in presence of 2ME2, its derivative STX564 or 0.02% (*v*/v) DMSO at the indicated concentrations. A–D: Depicted is the relative expression of interferon-γ, interleukin-17, interleukin-2 and tumor necrosis factor-α 6 h after antigen exposure measured by quantitative PCR. Housekeeping gene: β-actin. Non-stimulated T cells (-MBP) were used as negative control. Means ± SEM of 4 (A–D) independent experiments; * < 0.05; ** < 0.01; *** < 0.001. Statistical significance was assessed using one-way ANOVA with Bonferroni corrections (A–D). Blue asterisks indicate significant differences between control (-MBP, 0 μM) and 2ME2 conditions, red asterisks between control (-MBP, 0 μM) and STX564 conditions and black asterisks between 2ME2 and STX564 at indicated concentrations (A–D).

Having demonstrated that STX564 potently and specifically inhibited Ca^2+^ entry *via* SOCE channels in human Jurkat T-lymphoma cells, in primary rat MBP-specific effector T cells (T_MBP_ cells), in primary human CD4+ T cells, as well as in nonsmall cell lung cancer H1299 cells, we then tested the effect of this compound and also of 2ME2 on signaling events downstream of Ca^2+^ entry in T_MBP_ cells. TCR/CD3-evoked pro-inflammatory cytokine expression was significantly reduced at ≥0.1 μM STX564 (Fig. 7). 2ME2, included for comparison, was effective at significantly higher concentrations, respectively (Fig. 7).

**Fig. 8:**
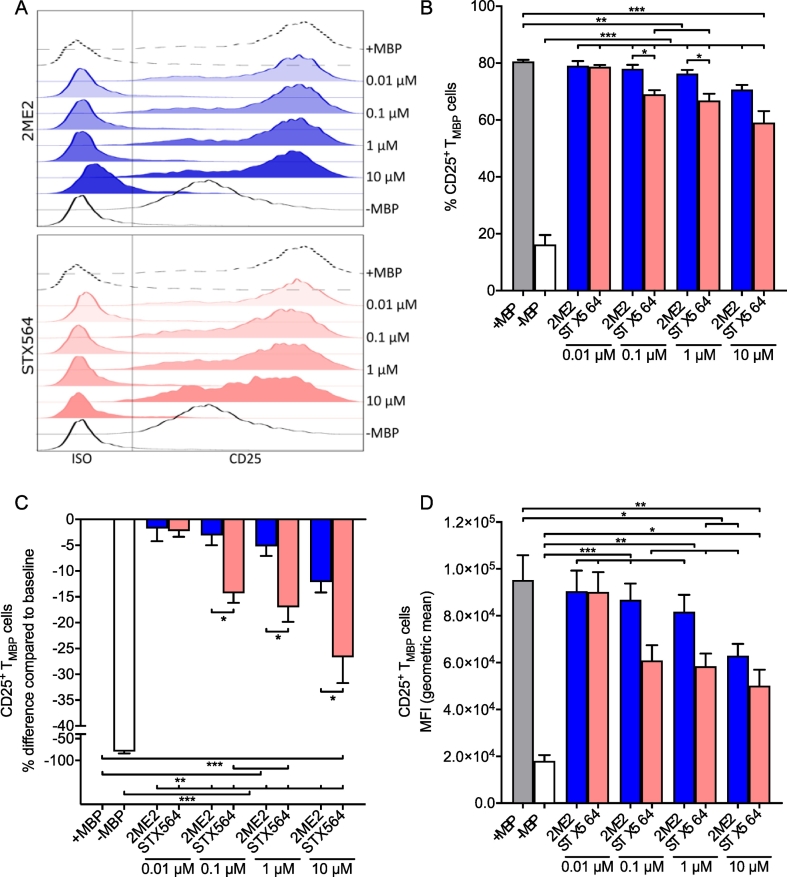
Fig. 6 STX564 inhibition of ORAI1 blocks antigen-dependent T cell activation. T_MBP_ cells were challenged *in vitro* as in Fig. 5A–D in presence of 2ME2 or STX564 at the indicated concentrations. Antigen-stimulated (+MBP) or non-stimulated (-MBP) T_MBP_ cells served as positive and negative control, respectively. A–D: Depicted are the histograms of CD25 expression on T_MBP_ cells (A), the quantification of the percentage of CD25^+^ T cells (B), the corresponding change in CD25 expression compared to baseline (C) and mean fluorescence intensity (MFI) based on the geometric mean (D). Means ± SEM of 5 independent experiments; * < 0.05; ** < 0.01; *** < 0.001. Statistical significance was assessed using one-way ANOVA with Bonferroni corrections.

These results were further corroborated by measuring protein expression of the surface activation marker CD25, which is strongly up-regulated upon T cell stimulation (Fig. 8). The up-regulation of CD25 was significantly reduced at ≥0.1 μM STX564, while a much higher concentration of 2ME2 was needed (Fig. 8), as quantified as the percentage of CD25-positive T cells (Fig. 8B), as the difference to MBP activation without inhibition (Fig. 8C), or, even more pronounced than percentage of cells, as mean fluorescence intensity of CD25-positive T cells (Fig. 8D). The latter shows, in addition to the reduced percentage of CD25-positive T cells, that there is also less expression of this important activation marker upon STX564 treatment. Taken together, these results suggest that STX564 mediated inhibition of the main SOCE components of T cells, ORAI and STIM, also results in substantial inhibition of major downstream events in effector T cell activation.

**Fig. 9:**
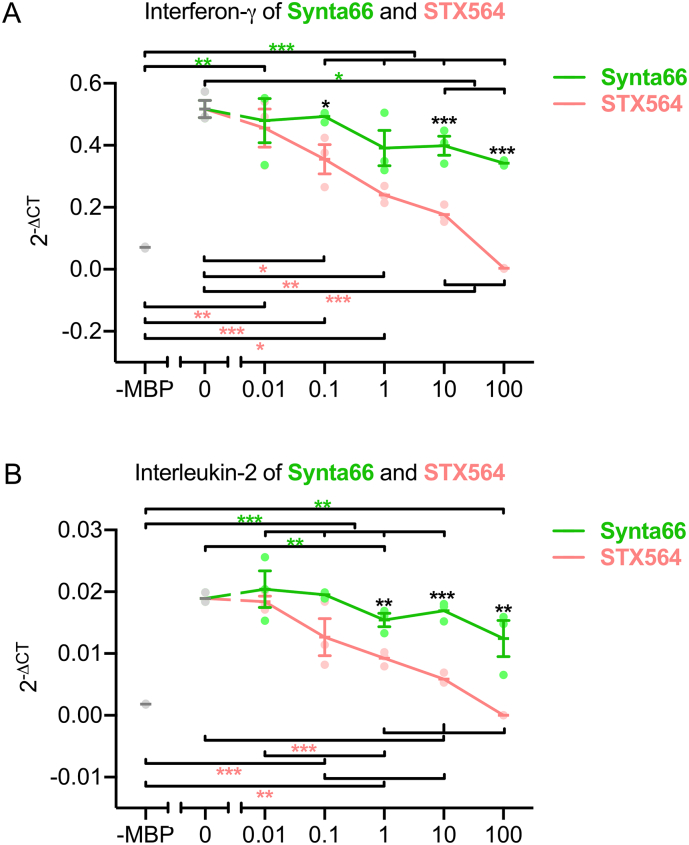
STX564 blocks cytokine production more efficiently than Synta66. T_MBP_ cells were challenged *in vitro* as in in Fig. 5A–D in presence of STX564 or Synta66 at the indicated concentrations. Antigen-stimulated (0 μM) or non-stimulated (-MBP) T_MBP_ cells served as positive and negative control, respectively. A–B: Depicted is the relative expression of interferon-γ and interleukin-2 6 h after antigen exposure measured by quantitative PCR. Housekeeping gene: β-actin. Means ± SEM of 3 independent experiments; * < 0.05; ** < 0.01; *** < 0.001. Statistical significance was assessed using one-way ANOVA with Bonferroni corrections. Green asterisks indicate significant differences between control (-MBP, 0 μM) and Synta66 conditions, red asterisks between control (-MBP, 0 μM) and STX564 conditions and black asterisks between Synta66 and STX564 at indicated concentrations.

Since ORAI channels are one of the two remaining targets of STX564, we next compared its antagonist potency regarding expression of interferon-γ and IL-2 in T_MBP_ cells with a known specific ORAI inhibitor, Synta 66. While Synta66 significantly antagonized interferon-γ at ≥10 μM, STX564 was effective already at ≥0.1 μM (Fig. 9A). Similar results were obtained for IL-2 expression (Fig. 9B).

**Fig. 10:**
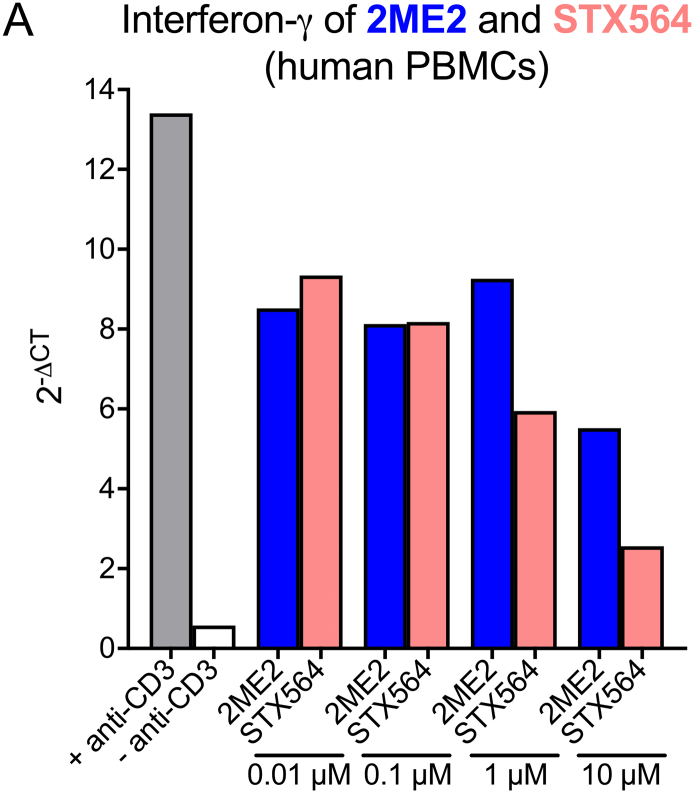
STX564 inhibition of ORAI1 blocks interferon-γ production of human PBMCs. Human PBMCs were challenged *in vitro* with anti-CD3 antibody in presence of 2ME2 or STX564 at the indicated concentrations. CD3-stimulated (+ anti-CD3) or non-stimulated (− anti-CD3) PBMCs served as positive and negative control, respectively. Depicted is the relative expression of interferon-γ 24 h after stimulation measured by quantitative PCR. Housekeeping gene: HPRT. Representative data from 2 healthy donors.

Finally, we went back to human PBMC and analyzed interferon-γ expression upon anti-CD3 stimulation. Antagonist effects were observed at ≥1 μM STX564, while an approx. 10-fold higher concentration of 2ME2 was necessary (Fig. 10).

## 4 Discussion

The central aspect of the current study was the development of a novel and efficient SOCE antagonist, through a chemical biology approach. The compounds evaluated in this study were previously investigated in cancer cell lines and in animal models of cancer [7,8,[14], [15], [16], [17], [18],^29^,^33^]. These studies showed that a 3-sulfamoyloxy group is advantageous over a 3-hydroxyl group and highly increases potency and bioavailability [15,29,33,34], a finding that was not confirmed in our study for Ca^2+^ entry inhibition in T cells *in vitro*. Nonetheless, a 3-sulfamoyloxy group increasing the potency of the compounds in an *in vivo* situation might be beneficial for potential administration of such compounds as drugs for *in vivo* treatment of autoimmune diseases, because 2-methoxy-3,17β-bis-sulfamoyloxy-estradiol was more potent than 2ME2 in earlier cancer-related studies [15,29,33,34]. Furthermore, it was shown in cancer cell lines that STX68 is more potent than STX139 that is more potent than 2ME2 [8]. For SOCE inhibition, STX139 is the most potent compound among these three compounds, whereas 2ME2 and STX68 exhibit a similar IC_50_ value (see Fig. 1). This suggests that the compounds do act on different targets in the different cell lines and disease models. Anti-proliferative and anti-angiogenic investigations in cancer cell lines revealed that methoxy and ethyl groups attached to C2 increase the potency of 2ME2 derivatives. Additionally, a sterically unhindered hydrogen bond acceptor attached to C17, like the cyanomethyl motif present in STX640, STX641, STX505, and STX564, also favors anti-proliferative and anti-angiogenic effects. These compounds belong to the most potent compounds for inhibiting SOCE in T cells in the current study, indicating that a sterically unhindered hydrogen bond acceptor at C17 causes the compounds to be more potent in cancer cell lines as well as in T cell Ca^2+^ signaling.

How does STX564 compare to Synta66? Both compounds inhibit SOCE in intact cells (see Fig. 5C-E). Despite Synta66 inhibiting ORAI1 in various cells (*e.g.* mast cells [35], T cells [36], platelets [37]), the patent WO2005/009954A2 (compound 66) reports that Synta66 also inhibits K_V_1.3 channels. However, Di Sabatino et al. [36] showed that 10 μM Synta66 inhibits alpha-dendrotoxin sensitive K_V_ channels, likely K_V_1.1, K_V_1.2 and/or K_V_1.6 [38], only to a minor degree (<10%). However, the K_V_ channel in human T cells, including Jurkat T cells, relevant for Ca^2+^ entry is K_V_1.3 as it helps to maintain Ca^2+^ influx by balancing entry of positive charges by K^+^ efflux [31,32,39]. For the new inhibitor STX564 we showed in patch clamp recordings that it does not inhibit the K_V_1.3 channel in Jurkat T cells. Thus, regarding inhibition of K_V_ channels STX564 appears more specific than Synta66. Regarding pro-inflammatory cytokine expression in rat effector T_MBP_ cells, an approx. 100-fold higher antagonist potency was observed for STX564 over Synta66, especially for INF-γ expression.

We clearly demonstrated that the hormone estradiol, its non-estrogenic metabolite 2ME2, and its synthetic analogue STX564 inhibit Ca^2+^ entry in T cells upon SOCE induction. As Ca^2+^ signaling and particularly a sustained increase of [Ca^2+^]_i_ is essential for downstream signaling events of T cell activation, inhibition of Ca^2+^ entry is likely the mechanism by which 2ME2 decreases NFAT translocation, T cell proliferation and cytokine production [12] as well as arresting the G2/M phase of the cell cycle [40]. Among the fast mechanisms potentially involved in elevated [Ca^2+^]_i_ upon exposure to STX564, we excluded experimentally (i) inhibition of IP_3_ evoked Ca^2+^ release, (ii) inhibition of SERCA activity, and (iii) inhibition of K_V_ as discussed above [41].

While it was shown that estradiol activates (i) Na^+^/K^+^ ATPases in human and mouse cardiac myocytes [42,43] (ii) TRPA1 and TRPV1 channels [44], and (iii) SERCA pumps in porcine and human heart arteries [42,45] as well as in rat muscle cells [46], it is unknown whether 2ME2 and its derivatives exert similar effects on these channels or pumps. Moreover, differential effects of estradiol on several K^+^ channels were described, *e.g.* activation [47,48] or inhibition [49]. Again, at least the 2ME2 derivative STX564 neither affected K_V_ channels nor SERCA pumps.

Since the current study showed a very rapid decrease in [Ca^2+^]_i_ upon STX564 administration, this effect clearly is not mediated by regulation of gene expression. However, steroid hormones and their derivatives were investigated in this study making estrogen receptors a potential target [50]. Estrogen receptors are divided into two subtypes, namely estrogen receptor α and β, both of which are expressed in T cells and are considered to mediate pro- as well as anti-inflammatory responses [50,51]. Modulation of gene expression involves the synthesis and modification of mRNA, the formation of proteins as well as the transport of proteins to their target compartment. This process takes about 7.5 to 30 min for the fastest known genomic actions of steroids (reviewed by [52]). Actions evoked by estradiol include downregulation of IL-2 receptor expression in murine thymocytes [53,54], modulation of secretion of interferon-γ in Th1 cells and interleukin-4 in Th2 cells, but also tumor necrosis factor-α and interleukin-17 secretion [53,54], which was controversially discussed by Khan and Ahmed (2015) [55]. Finally, estradiol also seems to increase the expression level of SERCA pumps in porcine heart arteries [45]. However, the current study provides good evidence that the inhibitory effect by 2ME2 and its derivatives is likely not due to involvement of estrogen receptors, as (i) the antagonist effects seen on ORAI channels occur immediately, and (ii) 2ME2 exhibits a 2.5-fold higher potency compared to estradiol in inhibiting Ca^2+^ entry, but binds with about 500-fold less affinity to estrogen receptors [9].

In conclusion, we characterized the 2ME2 derivative STX564 as a novel potent and specific SOCE inhibitor affecting neither Ca^2+^ release channels, SERCA pumps nor K_V_ channels. This compound should serve as an attractive new chemical biology tool for future studies on SOCE, but also in autoimmune disease models.

## Data and materials availability

Source data are available from the corresponding author upon request.

## Funding

This work was supported by the 10.13039/501100001659Deutsche Forschungsgemeinschaft (DFG), Germany, (project number 335447717; SFB1328, project A01 to AHG and AF, project A02 to B-PD, project A14 to ET; OD 87/1-1, OD 87/3-1 and SFB TRR 274/1 2020 – 408885537 to FO and AF) and by the Hertie Stiftung, Germany (grant 0070/120 to AHG, AF, BVLP). BVLP is a 10.13039/100010269Wellcome Trust Senior Investigator (grant 101010; Wellcome Trust, UK).

## CRediT authorship contribution statement

**Anke Löhndorf:** Conceptualization, Methodology, Validation, Formal analysis, Investigation, Data curation, Writing – original draft, Writing – review & editing, Visualization, Supervision, Project administration. **Leon Hosang:** Methodology, Formal analysis, Investigation, Data curation, Writing – original draft, Writing – review & editing, Visualization. **Wolfgang Dohle:** Investigation, Resources, Writing – review & editing. **Francesca Odoardi:** Conceptualization, Methodology, Validation, Investigation, Writing – original draft, Writing – review & editing, Supervision, Project administration. **Sissy-Alina Waschkowski:** Formal analysis, Investigation, Data curation, Writing – review & editing. **Anette Rosche:** Formal analysis, Investigation, Data curation. **Andreas Bauche:** Formal analysis, Investigation, Data curation. **Riekje Winzer:** Investigation. **Eva Tolosa:** Investigation. **Sabine Windhorst:** Investigation. **Stephen Marry:** Methodology, Formal analysis, Investigation, Data curation, Writing – review & editing. **Alexander Flügel:** Conceptualization, Validation, Writing – original draft, Writing – review & editing, Supervision, Project administration, Funding acquisition. **Barry V.L. Potter:** Conceptualization, Writing – original draft, Writing – review & editing, Supervision, Project administration, Funding acquisition. **Björn-Philipp Diercks:** Methodology, Formal analysis, Investigation, Data curation, Writing – review & editing. **Andreas H. Guse:** Conceptualization, Validation, Writing – original draft, Writing – review & editing, Supervision, Project administration, Funding acquisition.

## Declaration of competing interest

The authors declare no competing interest.
